# Supplementary material for: Modeling glioblastoma heterogeneity as a dynamic network of cell states
Source: Mol Syst Biol. 2021 Sep 16;17(9):e10105. doi: 10.15252/msb.202010105 (PMC8444284; doi:10.15252/msb.202010105)
Supplement: Supplementary file 6 — Source Data for Figure 5 [file MSB-17-e10105-s004.zip › Figure5A_sourcedata/GSEA_3071/nosigdb_stateI.GseaPreranked.1622795145664/DOROSZKO_U3013MG_CLUSTER_2.html]

Details for gene set DOROSZKO\_U3013MG\_CLUSTER\_2[GSEA]

|  || Dataset | state13071 |
| Phenotype | NoPhenotypeAvailable |
| Upregulated in class | na\_neg |
| GeneSet | DOROSZKO\_U3013MG\_CLUSTER\_2 |
| Enrichment Score (ES) | -0.6225156 |
| Normalized Enrichment Score (NES) | -3.5246623 |
| Nominal p-value | 0.0 |
| FDR q-value | 0.0 |
| FWER p-Value | 0.0 |
Table: GSEA Results Summary

  

Fig 1: Enrichment plot: DOROSZKO\_U3013MG\_CLUSTER\_2      
 Profile of the Running ES Score & Positions of GeneSet Members on the Rank Ordered List

  

| PROBE | GENE SYMBOL | GENE\_TITLE | RANK IN GENE LIST | RANK METRIC SCORE | RUNNING ES | CORE ENRICHMENT || 1 | UACA |  |  | 296 | 0.679 | -0.0820 | No |
| 2 | NUCB2 |  |  | 637 | 0.439 | -0.1795 | No |
| 3 | CAPS |  |  | 1367 | 0.258 | -0.3951 | No |
| 4 | CCDC34 |  |  | 1376 | 0.255 | -0.3951 | No |
| 5 | TTK |  |  | 1620 | -0.270 | -0.4652 | No |
| 6 | BUB3 |  |  | 1687 | -0.278 | -0.4823 | No |
| 7 | TEDC1 |  |  | 1698 | -0.279 | -0.4826 | No |
| 8 | KIF2C |  |  | 1735 | -0.284 | -0.4906 | No |
| 9 | CCNB2 |  |  | 1757 | -0.288 | -0.4941 | No |
| 10 | MIS18BP1 |  |  | 1764 | -0.289 | -0.4932 | No |
| 11 | APOLD1 |  |  | 1781 | -0.291 | -0.4952 | No |
| 12 | TROAP |  |  | 1821 | -0.296 | -0.5040 | No |
| 13 | CENPQ |  |  | 1860 | -0.300 | -0.5125 | No |
| 14 | BCL2L12 |  |  | 1918 | -0.307 | -0.5266 | No |
| 15 | KPNA2 |  |  | 1944 | -0.312 | -0.5311 | No |
| 16 | SPDL1 |  |  | 1968 | -0.314 | -0.5349 | No |
| 17 | PARPBP |  |  | 2026 | -0.322 | -0.5489 | No |
| 18 | DDX39A |  |  | 2043 | -0.324 | -0.5506 | No |
| 19 | PHIP |  |  | 2162 | -0.345 | -0.5826 | No |
| 20 | FAM83D |  |  | 2198 | -0.351 | -0.5897 | No |
| 21 | POC1A |  |  | 2199 | -0.351 | -0.5863 | No |
| 22 | MXD3 |  |  | 2201 | -0.352 | -0.5832 | No |
| 23 | PLK1 |  |  | 2206 | -0.353 | -0.5811 | No |
| 24 | CENPA |  |  | 2220 | -0.357 | -0.5815 | No |
| 25 | SOGA1 |  |  | 2234 | -0.360 | -0.5820 | No |
| 26 | BCL7C |  |  | 2274 | -0.366 | -0.5901 | No |
| 27 | TUBB4B |  |  | 2291 | -0.370 | -0.5914 | No |
| 28 | CKS1B |  |  | 2332 | -0.380 | -0.5997 | No |
| 29 | BUB1B |  |  | 2335 | -0.381 | -0.5966 | No |
| 30 | CKAP2 |  |  | 2357 | -0.385 | -0.5992 | No |
| 31 | ZWILCH |  |  | 2358 | -0.385 | -0.5955 | No |
| 32 | CENPW |  |  | 2402 | -0.393 | -0.6046 | No |
| 33 | BUB1 |  |  | 2416 | -0.396 | -0.6047 | No |
| 34 | WEE1 |  |  | 2426 | -0.397 | -0.6036 | No |
| 35 | CKS2 |  |  | 2466 | -0.404 | -0.6114 | No |
| 36 | AURKA |  |  | 2467 | -0.404 | -0.6075 | No |
| 37 | KIF11 |  |  | 2488 | -0.410 | -0.6096 | No |
| 38 | SPAG5 |  |  | 2519 | -0.418 | -0.6145 | No |
| 39 | RACGAP1 |  |  | 2522 | -0.419 | -0.6111 | No |
| 40 | CACYBP |  |  | 2536 | -0.424 | -0.6109 | No |
| 41 | CDC20 |  |  | 2540 | -0.425 | -0.6078 | No |
| 42 | NCAPD2 |  |  | 2563 | -0.431 | -0.6102 | No |
| 43 | CDK5RAP2 |  |  | 2564 | -0.431 | -0.6061 | No |
| 44 | ANP32B |  |  | 2593 | -0.438 | -0.6103 | No |
| 45 | HIRIP3 |  |  | 2635 | -0.449 | -0.6182 | Yes |
| 46 | MASTL |  |  | 2646 | -0.452 | -0.6169 | Yes |
| 47 | EXOSC8 |  |  | 2659 | -0.456 | -0.6161 | Yes |
| 48 | CCNA2 |  |  | 2667 | -0.462 | -0.6138 | Yes |
| 49 | SKA2 |  |  | 2672 | -0.464 | -0.6105 | Yes |
| 50 | PGP |  |  | 2681 | -0.468 | -0.6084 | Yes |
| 51 | MND1 |  |  | 2682 | -0.468 | -0.6039 | Yes |
| 52 | KIF22 |  |  | 2683 | -0.468 | -0.5994 | Yes |
| 53 | CDCA8 |  |  | 2695 | -0.470 | -0.5982 | Yes |
| 54 | ANLN |  |  | 2702 | -0.472 | -0.5955 | Yes |
| 55 | NEURL1B |  |  | 2704 | -0.472 | -0.5913 | Yes |
| 56 | PRC1 |  |  | 2705 | -0.472 | -0.5867 | Yes |
| 57 | CENPN |  |  | 2708 | -0.474 | -0.5828 | Yes |
| 58 | MELK |  |  | 2709 | -0.474 | -0.5783 | Yes |
| 59 | KNSTRN |  |  | 2722 | -0.478 | -0.5773 | Yes |
| 60 | NDC80 |  |  | 2723 | -0.478 | -0.5727 | Yes |
| 61 | YWHAH |  |  | 2725 | -0.479 | -0.5684 | Yes |
| 62 | RPA3 |  |  | 2730 | -0.480 | -0.5650 | Yes |
| 63 | SHCBP1 |  |  | 2733 | -0.480 | -0.5610 | Yes |
| 64 | CENPF |  |  | 2761 | -0.490 | -0.5643 | Yes |
| 65 | ACTL6A |  |  | 2762 | -0.492 | -0.5596 | Yes |
| 66 | NUF2 |  |  | 2781 | -0.497 | -0.5603 | Yes |
| 67 | DTYMK |  |  | 2798 | -0.501 | -0.5602 | Yes |
| 68 | LSM5 |  |  | 2820 | -0.509 | -0.5616 | Yes |
| 69 | ASF1B |  |  | 2824 | -0.510 | -0.5576 | Yes |
| 70 | CDCA3 |  |  | 2829 | -0.513 | -0.5539 | Yes |
| 71 | CDKN2D |  |  | 2832 | -0.514 | -0.5496 | Yes |
| 72 | SPC24 |  |  | 2836 | -0.514 | -0.5456 | Yes |
| 73 | HMGB3 |  |  | 2839 | -0.516 | -0.5412 | Yes |
| 74 | TOP2A |  |  | 2842 | -0.517 | -0.5369 | Yes |
| 75 | CEP55 |  |  | 2852 | -0.522 | -0.5346 | Yes |
| 76 | CKAP2L |  |  | 2857 | -0.523 | -0.5307 | Yes |
| 77 | ECT2 |  |  | 2858 | -0.523 | -0.5257 | Yes |
| 78 | NUDT1 |  |  | 2859 | -0.524 | -0.5207 | Yes |
| 79 | TRIP13 |  |  | 2865 | -0.526 | -0.5171 | Yes |
| 80 | RAD21 |  |  | 2866 | -0.527 | -0.5121 | Yes |
| 81 | HJURP |  |  | 2869 | -0.527 | -0.5076 | Yes |
| 82 | ANP32E |  |  | 2877 | -0.530 | -0.5047 | Yes |
| 83 | PTTG1 |  |  | 2895 | -0.540 | -0.5046 | Yes |
| 84 | CDKN2C |  |  | 2903 | -0.544 | -0.5014 | Yes |
| 85 | MT2A |  |  | 2910 | -0.547 | -0.4980 | Yes |
| 86 | FAM111A |  |  | 2917 | -0.552 | -0.4945 | Yes |
| 87 | ARHGAP11A |  |  | 2921 | -0.556 | -0.4901 | Yes |
| 88 | ATAD5 |  |  | 2935 | -0.563 | -0.4886 | Yes |
| 89 | PSRC1 |  |  | 2944 | -0.569 | -0.4855 | Yes |
| 90 | CENPH |  |  | 2945 | -0.569 | -0.4800 | Yes |
| 91 | KIF4A |  |  | 2953 | -0.574 | -0.4766 | Yes |
| 92 | TACC3 |  |  | 2956 | -0.575 | -0.4717 | Yes |
| 93 | KIF23 |  |  | 2961 | -0.579 | -0.4674 | Yes |
| 94 | PIMREG |  |  | 2971 | -0.583 | -0.4645 | Yes |
| 95 | NCAPH |  |  | 2974 | -0.585 | -0.4595 | Yes |
| 96 | GGH |  |  | 2975 | -0.585 | -0.4538 | Yes |
| 97 | POP7 |  |  | 2980 | -0.588 | -0.4494 | Yes |
| 98 | SMC2 |  |  | 2984 | -0.592 | -0.4446 | Yes |
| 99 | SKA3 |  |  | 2985 | -0.592 | -0.4390 | Yes |
| 100 | KNL1 |  |  | 2986 | -0.592 | -0.4333 | Yes |
| 101 | TPX2 |  |  | 3006 | -0.606 | -0.4332 | Yes |
| 102 | RTKN2 |  |  | 3020 | -0.613 | -0.4312 | Yes |
| 103 | AURKB |  |  | 3056 | -0.639 | -0.4355 | Yes |
| 104 | PHF19 |  |  | 3060 | -0.643 | -0.4302 | Yes |
| 105 | LMNB2 |  |  | 3061 | -0.644 | -0.4241 | Yes |
| 106 | JPT1 |  |  | 3062 | -0.644 | -0.4179 | Yes |
| 107 | NCAPD3 |  |  | 3064 | -0.646 | -0.4120 | Yes |
| 108 | ALYREF |  |  | 3068 | -0.649 | -0.4067 | Yes |
| 109 | SAC3D1 |  |  | 3073 | -0.655 | -0.4016 | Yes |
| 110 | FANCI |  |  | 3074 | -0.656 | -0.3953 | Yes |
| 111 | C21orf58 |  |  | 3078 | -0.659 | -0.3899 | Yes |
| 112 | GTSE1 |  |  | 3083 | -0.663 | -0.3847 | Yes |
| 113 | TMPO |  |  | 3088 | -0.666 | -0.3795 | Yes |
| 114 | SAP30 |  |  | 3096 | -0.671 | -0.3752 | Yes |
| 115 | MAD2L1 |  |  | 3099 | -0.675 | -0.3693 | Yes |
| 116 | RFC3 |  |  | 3108 | -0.683 | -0.3652 | Yes |
| 117 | KIFC1 |  |  | 3110 | -0.684 | -0.3589 | Yes |
| 118 | DBF4 |  |  | 3117 | -0.690 | -0.3541 | Yes |
| 119 | LMNB1 |  |  | 3121 | -0.692 | -0.3484 | Yes |
| 120 | PRR11 |  |  | 3125 | -0.698 | -0.3426 | Yes |
| 121 | SMC4 |  |  | 3126 | -0.699 | -0.3359 | Yes |
| 122 | CENPK |  |  | 3127 | -0.700 | -0.3292 | Yes |
| 123 | GMNN |  |  | 3140 | -0.719 | -0.3259 | Yes |
| 124 | KIF15 |  |  | 3142 | -0.723 | -0.3192 | Yes |
| 125 | BTG3 |  |  | 3148 | -0.727 | -0.3138 | Yes |
| 126 | CDK1 |  |  | 3149 | -0.727 | -0.3068 | Yes |
| 127 | MKI67 |  |  | 3158 | -0.738 | -0.3021 | Yes |
| 128 | ORC6 |  |  | 3159 | -0.738 | -0.2950 | Yes |
| 129 | TK1 |  |  | 3162 | -0.745 | -0.2885 | Yes |
| 130 | DNAJC9 |  |  | 3167 | -0.747 | -0.2825 | Yes |
| 131 | USP1 |  |  | 3183 | -0.762 | -0.2797 | Yes |
| 132 | BRCA1 |  |  | 3185 | -0.765 | -0.2727 | Yes |
| 133 | CDCA2 |  |  | 3189 | -0.767 | -0.2662 | Yes |
| 134 | PSMC3IP |  |  | 3194 | -0.772 | -0.2600 | Yes |
| 135 | CIP2A |  |  | 3199 | -0.782 | -0.2537 | Yes |
| 136 | SGO1 |  |  | 3202 | -0.783 | -0.2468 | Yes |
| 137 | BIRC5 |  |  | 3208 | -0.793 | -0.2407 | Yes |
| 138 | DIAPH3 |  |  | 3213 | -0.800 | -0.2342 | Yes |
| 139 | RRM1 |  |  | 3215 | -0.801 | -0.2269 | Yes |
| 140 | SPC25 |  |  | 3219 | -0.806 | -0.2200 | Yes |
| 141 | UBE2C |  |  | 3225 | -0.812 | -0.2137 | Yes |
| 142 | NUSAP1 |  |  | 3237 | -0.825 | -0.2091 | Yes |
| 143 | FOXM1 |  |  | 3238 | -0.826 | -0.2012 | Yes |
| 144 | TMSB15A |  |  | 3241 | -0.833 | -0.1938 | Yes |
| 145 | CCP110 |  |  | 3262 | -0.870 | -0.1915 | Yes |
| 146 | CENPM |  |  | 3269 | -0.881 | -0.1848 | Yes |
| 147 | CDCA5 |  |  | 3273 | -0.885 | -0.1772 | Yes |
| 148 | FEN1 |  |  | 3278 | -0.890 | -0.1699 | Yes |
| 149 | NSD2 |  |  | 3281 | -0.896 | -0.1619 | Yes |
| 150 | H2AFX |  |  | 3282 | -0.898 | -0.1533 | Yes |
| 151 | CDKN3 |  |  | 3293 | -0.916 | -0.1475 | Yes |
| 152 | ZWINT |  |  | 3296 | -0.925 | -0.1392 | Yes |
| 153 | UBE2T |  |  | 3330 | -0.979 | -0.1397 | Yes |
| 154 | MYBL2 |  |  | 3340 | -1.006 | -0.1328 | Yes |
| 155 | EZH2 |  |  | 3342 | -1.015 | -0.1233 | Yes |
| 156 | RAD51AP1 |  |  | 3345 | -1.030 | -0.1141 | Yes |
| 157 | PBK |  |  | 3354 | -1.046 | -0.1064 | Yes |
| 158 | ESCO2 |  |  | 3367 | -1.088 | -0.0996 | Yes |
| 159 | NCAPG |  |  | 3370 | -1.098 | -0.0897 | Yes |
| 160 | DHFR |  |  | 3387 | -1.159 | -0.0833 | Yes |
| 161 | HMGB2 |  |  | 3390 | -1.184 | -0.0726 | Yes |
| 162 | CLSPN |  |  | 3391 | -1.184 | -0.0612 | Yes |
| 163 | UBE2S |  |  | 3396 | -1.205 | -0.0509 | Yes |
| 164 | CENPU |  |  | 3398 | -1.230 | -0.0394 | Yes |
| 165 | PCLAF |  |  | 3409 | -1.265 | -0.0303 | Yes |
| 166 | ATAD2 |  |  | 3415 | -1.278 | -0.0195 | Yes |
| 167 | RRM2 |  |  | 3420 | -1.297 | -0.0083 | Yes |
| 168 | TYMS |  |  | 3436 | -1.391 | 0.0006 | Yes |
| 169 | HIST1H4C |  |  | 3493 | -2.248 | 0.0054 | Yes |
Table: GSEA details [plain text format]

  

Fig 2: DOROSZKO\_U3013MG\_CLUSTER\_2: Random ES distribution      
 Gene set null distribution of ES for **DOROSZKO\_U3013MG\_CLUSTER\_2**
